# Fatal noncardiogenic pulmonary edema related to nonionic, iso-osmolar iodine contrast medium: one case report

**DOI:** 10.1186/s12890-022-01908-0

**Published:** 2022-03-31

**Authors:** Kun-ming Yi, Xue Li

**Affiliations:** 1 Department of Radiology, Daping Hospital, Army Medical University, Chongqing, 400042 China; 2 Chongqing Clinical Research Centre of Imaging and Nuclear Medicine, Chongqing, 400042 China

**Keywords:** Acute pulmonary edema, Noncardiogenic pulmonary edema, Iodinated contrast medium, Iso-osmolar contrast medium

## Abstract

**Background:**

Noncardiogenic pulmonary edema (NCPE) is a rare and life-threatening allergy-like reaction to the intravascular injection of a nonionic radiographic agent. We first describe a very rare case of fatal NCPE after the intravenous injection of nonionic, iso-osmolar iodine contrast media.

Case presentation

A 55-year-old male patient was admitted to the hospital with esophageal cancer. After the intravenous administration of 100 mL iodixanol, the patient first exhibited digestive tract symptoms, including abdominal pain, diarrhea, and vomiting, with no dyspnea, rash, itching, or throat edema. He received anti-allergy treatment, but his symptoms did not improve; instead, he further developed pulmonary edema. Arterial blood gas analysis results were as follows: pH, 7.08; PO_2_, 70 mm Hg; PCO_2_, 40 mm Hg; and SaO_2_, 52%. Then, the patient received emergent tracheal intubation and ventilation to assist breathing, and he was transferred to the intensive care unit (ICU) for further treatment. In the ICU, the patient developed shock and respiratory and circulatory failure; therefore, he received shock resuscitation, acidosis correction, muscle relaxants to lower the work of breathing, and cardiotonic therapy. The patient eventually died. During the ICU period, emergency bedside color ultrasound showed a diffuse B line in both lungs, and the size of the cardiac cavity was normal, but the ventricular rate was extremely fast. Chest radiography showed pulmonary edema with a normal cardiac silhouette, and the brain natriuretic peptide (BNP) level was in the normal range.

**Conclusions:**

NCPE is a rare and critical allergy-like reaction to the use of a nonionic iso-osmolar radiocontrast contrast medium. Clinicians should pay very close attention to digestive tract manifestations during the medical observation of patients, as gastrointestinal manifestations may be the prodromal symptoms of NCPE caused by iso-osmolar contrast medium injection.

## Background

Pulmonary edema is a rare (0.001–0.008%) and life-threatening complication of intravascular contrast agent injection; however, pulmonary edema is common and observed in 10–20% of fatal reactions to contrast media injection [1]. Noncardiogenic pulmonary edema (NCPE) after intravenous administration of nonionic iodine contrast media (ICM) might occur in patients receiving high-osmolar or low-osmolar agents [1–4]. We herein report a rare case of NCPE with gastrointestinal symptoms as the initial symptom following the intravascular injection of iso-osmolar ICM.

## Case presentation

**Fig. 1:**
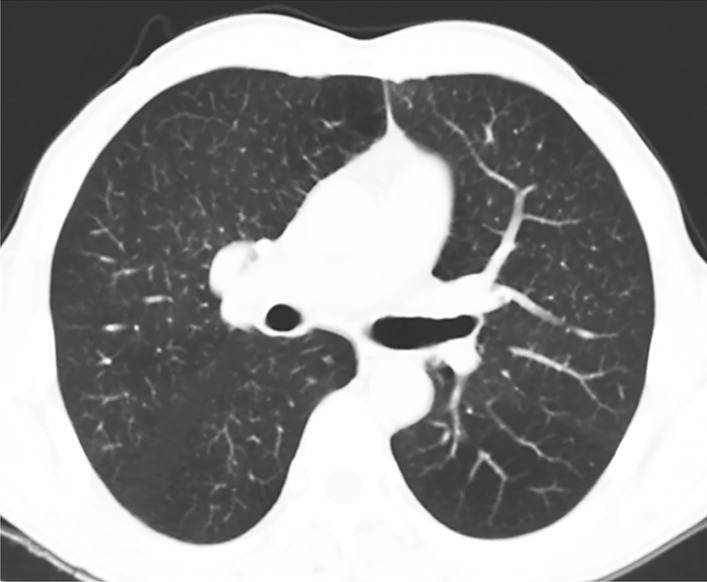
Chest CT scan showing no ground-glass exudation, patchy shadow, or consolidation in either lung before the incident

A 55-year-old man was admitted to the hospital due to middle esophageal cancer. The patient had no family medical history, hypertension, coronary heart disease, allergies, or ICM use history. On admission, his respiratory rate was 16 breaths per min, his heart rate was 52 beats per min, and his blood pressure was 102/72 mm Hg. He also underwent coronary computed tomography angiography (CTA) and a chest-abdominal enhanced computed tomography (CT) scan. During this scan, the patient received 100 mL iodixanol (Visipaque 320, Hengrui Medicine, Jiang Su, China), which is a nonionic, iso-osmolar ICM. Coronary CTA detected multiple-vessel disease with localized mild coronary narrowing (30–40% diameter stenosis) located at the proximal and middle sections of the right coronary artery, left anterior descending artery, and left circumflex artery. Chest CT scan showed middle esophageal cancer without exudation, patchy shadow, or consolidation in both lungs (Fig. 1). There were no abnormal findings on the abdominal CT scan. Thirty-five minutes later, when the patient returned to the thoracic surgery ward, he developed abdominal pain, diarrhea, and vomiting, with no dyspnea, rash, itching, wheezing, or throat edema. He received dexamethasone sodium phosphate 10 mg intravenously, intravenous rehydration, and oxygen inhalation to treat a suspected allergy-like reaction caused by ICM. Epinephrine was not used.

**Fig. 2:**
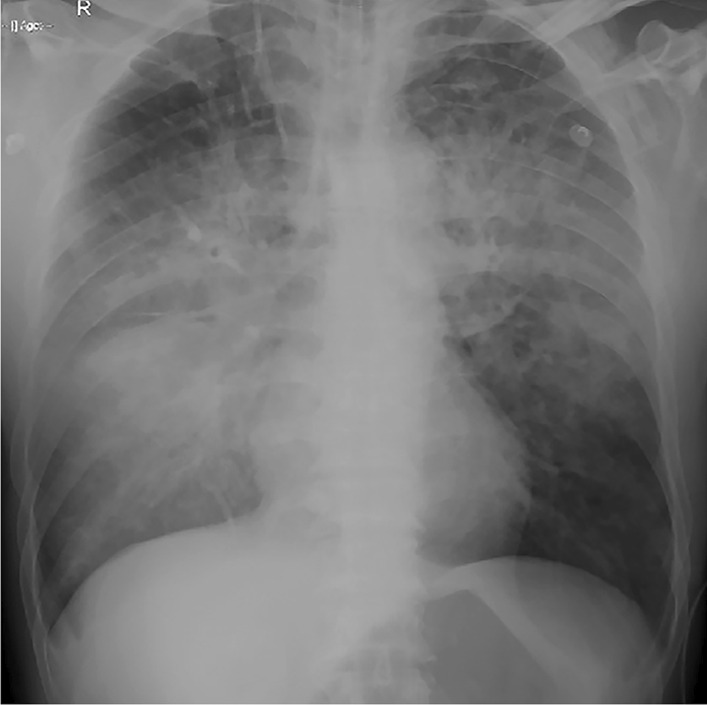
Emergency bedside chest radiography showing features of pulmonary edema with a normal cardiac silhouette during the ICU period

One hour after the onset of symptoms, he developed dyspnea and wheezing. Widespread moist rales and wheezes occurred in both lung fields. His respiratory rate was 35–40 breaths/min, his heart rate was 160–170 beats/min, and his blood pressure was 107/76 mm Hg. Arterial blood gas analysis results were as follows: pH, 7.08; PO_2_, 70 mm Hg; PCO_2_, 40 mm Hg; Lac, 4.7 mmol/L; and SaO_2_, 52%. Emergent tracheal intubation was immediately performed, and a ventilator was used to assist breathing. Meanwhile, the patient also received diuretic treatment due to pulmonary edema. The patient was transferred to the intensive care unit (ICU) for further treatment. The electrocardiogram showed sinus tachycardia (heart rate was 170 beats/min); blood pressure was 124/63 mm Hg, and the respiratory rate was 40 breaths/min. Emergency bedside color ultrasound showed a diffuse B line in both lungs, and the size of the cardiac cavity was normal, but the ventricular rate was extremely fast. Emergency bedside chest radiography suggested pulmonary edema with a normal cardiac silhouette (Fig. 2). Repeated arterial blood gas analysis results were as follows: pH, 7.05; PO_2_, 92 mm Hg; PCO_2_, 59 mm Hg; and Lac, 4.7 mmol/L (FIO_2_ 100%). Blood routine test results were as follows: Hb 207 g/L, and PLT 301 × 10^9^/L.

Two hours after the onset of symptoms, the patient returned to shock but had tachycardia, and he was given rapid fluid resuscitation. Worse, the patient's symptoms were not significantly improved, and his blood pressure became unstable. He received norepinephrine administration (1–1.5 µg/kg/min) through the right internal jugular central venous catheter. The oxygen saturation of the patient decreased six hours after the onset of symptoms, positive end-expiratory pressure was increased to 10 cm H_2_O, and he also received muscle relaxants to lower the work of breathing and levosimendan combined with deslanoside for cardiotonic therapy. The patient's condition further deteriorated; ten hours after the intravenous injection of contrast, the patient died. Brain natriuretic peptide (BNP), measured 6 h after the onset of symptoms, was normal.

## Discussion

In this case, the patient's BNP level was in the normal range, and there was no electrical or biological evidence of acute myocardial infarction. The chest radiography showed features of pulmonary edema with a normal cardiac silhouette. Consequently, we considered that the causal factor of the patient's death was more likely to be acute noncardiogenic pulmonary edema (NCPE) than cardiogenic pulmonary edema (CPE). CPE is caused by the high osmotic load of intravenous contrast agents (higher osmolar, higher viscous, and ionic contrast media), especially for patients with pre-existing cardiovascular compromise [2].

The major respiratory adverse reactions to intravascular use of ICM include dyspnea, bronchospasm, apnea, pulmonary edema, and an increase in pulmonary arterial blood pressure. Acute NCPE is rare [5]. Various pathogeneses of contrast-induced, noncardiogenic pulmonary edema have been reported. Mediator release and complement activation resulting in endothelial damage could be the main pathogeneses. Endothelial injury causes an increase in microvascular permeability, which in turn leads to the accumulation of fluid in the lung, and the leakage of fluids from the circulation results in an increased hemoglobin concentration and packed-cell volume [2, 5, 6]. A direct irritant effect of the ICM on the lungs is another theory [2]. Contrast media can activate the complement system to release a large number of substances that cause vasodilatation, increased vascular permeability, edema, increased smooth muscle cell contraction and precipitating bronchospasm, and increased mucus secretion in airways [7]. We believe that the mechanism of the contrast agent causing gastrointestinal symptoms is similar to that causing respiratory symptoms. Thus, gastrointestinal damage may indirectly indicate respiratory injury.

For patients who develop NCPE after intravenous injection of a contrast agent, the basic treatment guidelines are the ABCs of the airway, breathing, and circulation. The primary emergency treatment is the administration of oxygen with continuous positive airway pressure or invasive ventilation with positive end-expiratory pressure, together with fluid resuscitation to increase left ventricular preload [6]. However, treatment with diuretics or vasodilators should be avoided because these could cause disease deterioration [2]. Epinephrine is recommended as the preferred drug for treating anaphylaxis and anaphylactoid reactions by the World Allergy Organization [8]. Furthermore, the use of hydrocortisone may reduce the inflammatory response and endothelial injury [3, 6]. Timely use of extracorporeal membrane oxygenation (ECMO) can be lifesaving in the treatment of acute, medically nonresponsive severe cardiopulmonary failure after intravascular injection of contrast media [9].

In conclusion, we report the first case of NCPE caused by intravascular injection of iso-osmolar ICM. We emphasize that the intravascular administration of nonionic iso-osmolar ICM may cause fatal NCPE and that initial gastrointestinal manifestations may be the prodromal symptoms of NCPE. In addition, timely distinguishing NCPE from CPE is the key to treatment in an emergency context, and the medical history of the patient’s pre-existing cardiovascular compromise, laboratory tests with BNP, and chest CT and chest radiography can help to differentiate NCPE from CPE.

## Data Availability

The datasets used during the current study are available from the corresponding author on reasonable request.

## Abbreviations

ICM: Iodine contrast medium
CT: Computed tomography
CTA: Computed tomography angiography
pH: Potential of hydrogen
PCO_2_: Arterial partial pressure of carbon dioxide
PO_2_: Arterial partial pressure of oxygen
SaO_2_: Oxygen saturation
Lac: Lactate
Hb: Hemoglobin
PLT: Platelet
ICU: Intensive care unit
FIO_2_: Fraction of inspiring oxygen
BNP: Brain natriuretic peptide
NCPE: Noncardiogenic pulmonary edema
CPE: Cardiogenic pulmonary edema
ECMO: Extracorporeal membrane oxygenation

## References

[1] Hauggaard A (1996). Non-cardiogenic pulmonary oedema after intravenous administration of non-ionic contrast media. Acta Radiol.

[2] Paul RE, George G (2002). Fatal non-cardiogenic pulmonary oedema after intravenous non-ionic radiographic contrast. Lancet.

[3] Pincet L, Lecca G (2018). Acute pulmonary edema induced by non-ionic low-osmolar radiographic contrast media. Open Access Emerg Med.

[4] Kang MH, Nah JC (2013). A delayed, unusual non-cardiogenic pulmonary edema after intravascular administration of non-ionic, low osmolar radiocontrast media for coronary angiography. Korean Circ J.

[5] Morcos SK (2003). Review article. Effects of radiographic contrast media on the lung. Br J Radiol.

[6] Pasternak JJ, Williamson EE (2012). Clinical pharmacology, uses, and adverse reactions of iodinated contrast agents: a primer for the non-radiologist. Mayo Clin Proc.

[7] Schopp JG, Iyer RS, Wang CL, Petscavage JM, Paladin AM, Bush WH (2013). Allergic reactions to iodinated contrast media: premedication considerations for patients at risk. Emerg Radiol.

[8] Kemp SF, Lockey RF, Simons FE (2008). Epinephrine: the drug of choice for anaphylaxis a statement of the World Allergy Organization. Allergy.

[9] Guru PK, Bohman JK, Fleming CJ, Tan HL, Sanghavi DK, Moraes AG (2016). Severe acute cardiopulmonary failure related to gadobutrol magnetic resonance imaging contrast reaction: successful resuscitation with extracorporeal membrane oxygenation. Mayo Clin Proc.

